# 4,4′,6,6′-Tetra-*tert*-butyl-2,2′-[butane-1,4-diylbis(nitrilo­methanylyl­idene)]diphenol

**DOI:** 10.1107/S1600536811041614

**Published:** 2011-10-12

**Authors:** Jia Ti Tee, Norbani Abdullah, Hamid Khaledi

**Affiliations:** a Department of Chemistry, University of Malaya, 50603 Kuala Lumpur, Malaysia

## Abstract

The title compound, C_34_H_52_N_2_O_2_, is centrosymmetric, the mid-point of the central C—C bond being located on an inversion centre. Intra­molecular O—H⋯N and weak C—H⋯O hydrogen bonds are observed, but no significant inter­molecular inter­actions occur in the crystal structure.

## Related literature

For structures of some metal complexes of the title Schiff base, see: Doyle *et al.* (2007 ▶); Keizer *et al.* (2002*a* ▶,*b* ▶).
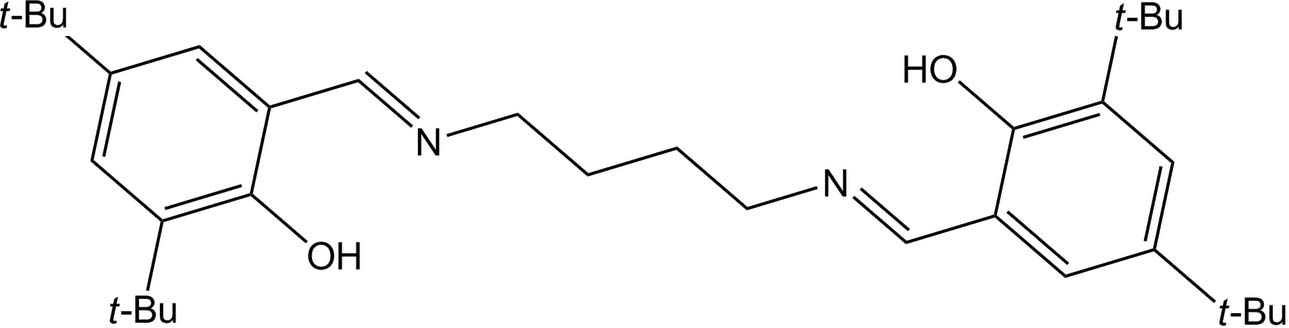


## Experimental

### 

#### Crystal data

C_34_H_52_N_2_O_2_
*M* *_r_* = 520.78
Monoclinic, 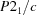

*a* = 19.1255 (4) Å
*b* = 9.5702 (2) Å
*c* = 8.6312 (1) Å
β = 90.383 (1)°
*V* = 1579.78 (5) Å^3^
*Z* = 2
Mo *K*α radiation
μ = 0.07 mm^−1^
*T* = 100 K
0.26 × 0.15 × 0.06 mm

#### Data collection

Bruker APEXII CCD diffractometer
Absorption correction: multi-scan (*SADABS*; Sheldrick, 1996 ▶) *T*_min_ = 0.983, *T*_max_ = 0.996
14602 measured reflections
3631 independent reflections
3039 reflections with *I* > 2σ(*I*)
*R*_int_ = 0.024

#### Refinement

*R*[*F* ^2^ > 2σ(*F* ^2^)] = 0.043
*wR*(*F* ^2^) = 0.114
*S* = 1.03
3631 reflections
181 parameters
H atoms treated by a mixture of independent and constrained refinement
Δρ_max_ = 0.30 e Å^−3^
Δρ_min_ = −0.16 e Å^−3^

### 

Data collection: *APEX2* (Bruker, 2007 ▶); cell refinement: *SAINT* (Bruker, 2007 ▶); data reduction: *SAINT*; program(s) used to solve structure: *SHELXS97* (Sheldrick, 2008 ▶); program(s) used to refine structure: *SHELXL97* (Sheldrick, 2008 ▶); molecular graphics: *X-SEED* (Barbour, 2001 ▶); software used to prepare material for publication: *SHELXL97* and *publCIF* (Westrip, 2010 ▶).

## Supplementary Material

Crystal structure: contains datablock(s) I, global. DOI: 10.1107/S1600536811041614/xu5350sup1.cif

Structure factors: contains datablock(s) I. DOI: 10.1107/S1600536811041614/xu5350Isup2.hkl

Additional supplementary materials:  crystallographic information; 3D view; checkCIF report

## Figures and Tables

**Table 1:** Hydrogen-bond geometry (Å, °)

*D*—H⋯*A*	*D*—H	H⋯*A*	*D*⋯*A*	*D*—H⋯*A*
O1—H1⋯N1	0.927 (16)	1.735 (17)	2.5840 (13)	150.8 (14)
C8—H8*B*⋯O1	0.98	2.29	2.9546 (16)	125
C9—H9*A*⋯O1	0.98	2.44	3.0720 (15)	122

**Fig. 1.**
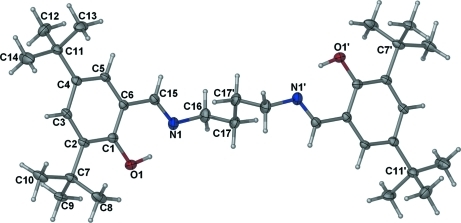
Molecular structure of the title compound with displacement ellipsoids at the 50% probability level. Hydrogen atoms are drawn as spheres of arbitrary radius.

## supplementary crystallographic information

### Comment

The title Schiff base has been displayed ambidentate ligation behavior towards metal ions (Doyle *et al.*, 2007; Keizer *et al.*, 2002*a*,*b*). Herein, wish to report the crystal structure of the free ligand, obtained through the condensation reaction of 3,5-di-*tert*-butyl-2-hydroxybenzaldehyde and 1,4-diaminobutane. The molecule lies across a crystallographic inversion centre. The imino group is almost coplanar with the phenyl ring [dihedral angle = 3.00 (13)] and adopts an *E* configuration. The hydroxyl group is engaged in an intramolecular O—H···N hydrogen bond with the imine group. Moreover, it acts as an acceptor in two intramolecular C—H···O hydrogen bonds (Table 1). The structure does not display any significant intermolecular interactions.

### Experimental

3,5-Di-*tert*-butyl-2-hydroxybenzaldehyde (5.86 g, 25 mmol) was dissolved in methanol (50 ml) in a round-bottomed flask fitted with a reflux condenser. The solution was heated, followed by portionwise addition of 1,4-diaminobutane (1.10 g; 12.5 mmol). The pale yellow solution formed was then gently refluxed for 3 h. The product obtained on cooling was recrystallized from ethanol at room temperature to give X-ray quality crystals of the title compound.

### Refinement

The C-bound H atoms were placed at calculated positions and refined as riding on their parent atoms, with C–H = 0.95 (aryl), 0.98 (methyl) and 0.99 (methylene) Å. The O-bound H atom was located in a difference Fourier map and refined freely. For all H atoms *U*_iso_(H) were set to 1.2–1.5*U*_eq_(carrier atom).

### Crystal data

**Table:**

C_34_H_52_N_2_O_2_	*F*(000) = 572
*M**_r_* = 520.78	*D*_x_ = 1.095 Mg m^−^^3^
Monoclinic, *P*2_1_/*c*	Mo *K*α radiation, λ = 0.71073 Å
Hall symbol: -P 2ybc	Cell parameters from 4857 reflections
*a* = 19.1255 (4) Å	θ = 2.4–30.3°
*b* = 9.5702 (2) Å	µ = 0.07 mm^−^^1^
*c* = 8.6312 (1) Å	*T* = 100 K
β = 90.383 (1)°	Plate, yellow
*V* = 1579.78 (5) Å^3^	0.26 × 0.15 × 0.06 mm
*Z* = 2	

### Data collection

**Table:**

Bruker APEXII CCD diffractometer	3631 independent reflections
Radiation source: fine-focus sealed tube	3039 reflections with *I* > 2σ(*I*)
graphite	*R*_int_ = 0.024
φ and ω scans	θ_max_ = 27.5°, θ_min_ = 2.1°
Absorption correction: multi-scan (*SADABS*; Sheldrick, 1996)	*h* = −24→24
*T*_min_ = 0.983, *T*_max_ = 0.996	*k* = −12→12
14602 measured reflections	*l* = −11→11

### Refinement

**Table:**

Refinement on *F*^2^	Primary atom site location: structure-invariant direct methods
Least-squares matrix: full	Secondary atom site location: difference Fourier map
*R*[*F*^2^ > 2σ(*F*^2^)] = 0.043	Hydrogen site location: inferred from neighbouring sites
*wR*(*F*^2^) = 0.114	H atoms treated by a mixture of independent and constrained refinement
*S* = 1.03	*w* = 1/[σ^2^(*F*_o_^2^) + (0.0533*P*)^2^ + 0.5079*P*] where *P* = (*F*_o_^2^ + 2*F*_c_^2^)/3
3631 reflections	(Δ/σ)_max_ < 0.001
181 parameters	Δρ_max_ = 0.30 e Å^−^^3^
0 restraints	Δρ_min_ = −0.16 e Å^−^^3^

### Special details

**Table:**

Geometry. All e.s.d.'s (except the e.s.d. in the dihedral angle between two l.s. planes) are estimated using the full covariance matrix. The cell e.s.d.'s are taken into account individually in the estimation of e.s.d.'s in distances, angles and torsion angles; correlations between e.s.d.'s in cell parameters are only used when they are defined by crystal symmetry. An approximate (isotropic) treatment of cell e.s.d.'s is used for estimating e.s.d.'s involving l.s. planes.
Refinement. Refinement of *F*^2^ against ALL reflections. The weighted *R*-factor *wR* and goodness of fit *S* are based on *F*^2^, conventional *R*-factors *R* are based on *F*, with *F* set to zero for negative *F*^2^. The threshold expression of *F*^2^ > σ(*F*^2^) is used only for calculating *R*-factors(gt) *etc*. and is not relevant to the choice of reflections for refinement. *R*-factors based on *F*^2^ are statistically about twice as large as those based on *F*, and *R*- factors based on ALL data will be even larger.

### Fractional atomic coordinates and isotropic or equivalent isotropic displacement parameters (Å^2^)

**Table:**

	*x*	*y*	*z*	*U*_iso_*/*U*_eq_	
O1	0.38365 (4)	0.98230 (9)	0.47339 (10)	0.0267 (2)	
H1	0.4145 (8)	0.9488 (17)	0.3998 (19)	0.040*	
N1	0.43079 (5)	0.86964 (11)	0.22286 (12)	0.0276 (2)	
C1	0.31824 (6)	0.96886 (11)	0.41401 (12)	0.0198 (2)	
C2	0.26044 (6)	1.01998 (11)	0.49629 (12)	0.0193 (2)	
C3	0.19476 (6)	1.00283 (11)	0.42796 (12)	0.0203 (2)	
H3	0.1553	1.0373	0.4824	0.024*	
C4	0.18292 (6)	0.93820 (12)	0.28429 (12)	0.0197 (2)	
C5	0.24129 (6)	0.88907 (11)	0.20702 (12)	0.0196 (2)	
H5	0.2353	0.8443	0.1096	0.024*	
C6	0.30834 (6)	0.90361 (11)	0.26843 (12)	0.0194 (2)	
C7	0.26927 (7)	1.09122 (12)	0.65518 (12)	0.0230 (3)	
C8	0.31935 (8)	1.21639 (13)	0.64423 (15)	0.0348 (3)	
H8A	0.3004	1.2849	0.5707	0.052*	
H8B	0.3651	1.1842	0.6085	0.052*	
H8C	0.3244	1.2599	0.7465	0.052*	
C9	0.29756 (7)	0.98400 (12)	0.77258 (13)	0.0254 (3)	
H9A	0.3430	0.9492	0.7377	0.038*	
H9B	0.2647	0.9058	0.7807	0.038*	
H9C	0.3030	1.0286	0.8741	0.038*	
C10	0.19963 (7)	1.14567 (14)	0.71697 (14)	0.0323 (3)	
H10A	0.2074	1.1915	0.8172	0.049*	
H10B	0.1672	1.0674	0.7300	0.049*	
H10C	0.1797	1.2130	0.6434	0.049*	
C11	0.10988 (6)	0.92199 (13)	0.21236 (13)	0.0246 (3)	
C12	0.09358 (7)	0.76642 (15)	0.19055 (17)	0.0357 (3)	
H12A	0.0932	0.7200	0.2918	0.053*	
H12B	0.1294	0.7237	0.1252	0.053*	
H12C	0.0477	0.7560	0.1407	0.053*	
C13	0.10838 (7)	0.99276 (15)	0.05301 (15)	0.0339 (3)	
H13A	0.0630	0.9757	0.0029	0.051*	
H13B	0.1457	0.9542	−0.0114	0.051*	
H13C	0.1154	1.0936	0.0656	0.051*	
C14	0.05296 (7)	0.9870 (2)	0.31222 (17)	0.0449 (4)	
H14A	0.0526	0.9413	0.4138	0.067*	
H14B	0.0074	0.9745	0.2615	0.067*	
H14C	0.0623	1.0870	0.3255	0.067*	
C15	0.36766 (6)	0.85464 (12)	0.17746 (13)	0.0225 (2)	
H15	0.3589	0.8100	0.0810	0.027*	
C16	0.48707 (6)	0.82786 (14)	0.11850 (16)	0.0312 (3)	
H16A	0.4676	0.7714	0.0324	0.037*	
H16B	0.5214	0.7697	0.1755	0.037*	
C17	0.52336 (6)	0.95676 (14)	0.05349 (15)	0.0294 (3)	
H17A	0.5653	0.9268	−0.0045	0.035*	
H17B	0.5393	1.0163	0.1407	0.035*	

### Atomic displacement parameters (Å^2^)

**Table:**

	*U*^11^	*U*^22^	*U*^33^	*U*^12^	*U*^13^	*U*^23^
O1	0.0244 (4)	0.0311 (5)	0.0246 (4)	−0.0036 (4)	−0.0030 (3)	−0.0032 (3)
N1	0.0240 (5)	0.0289 (6)	0.0299 (5)	−0.0012 (4)	0.0047 (4)	−0.0003 (4)
C1	0.0244 (6)	0.0152 (5)	0.0196 (5)	−0.0026 (4)	−0.0019 (4)	0.0027 (4)
C2	0.0299 (6)	0.0126 (5)	0.0155 (5)	0.0009 (4)	−0.0011 (4)	0.0015 (4)
C3	0.0273 (6)	0.0173 (5)	0.0163 (5)	0.0051 (4)	0.0019 (4)	0.0010 (4)
C4	0.0241 (6)	0.0183 (5)	0.0167 (5)	0.0011 (4)	−0.0012 (4)	0.0022 (4)
C5	0.0271 (6)	0.0169 (5)	0.0149 (5)	−0.0014 (4)	0.0005 (4)	−0.0011 (4)
C6	0.0245 (6)	0.0150 (5)	0.0188 (5)	−0.0013 (4)	0.0023 (4)	0.0014 (4)
C7	0.0365 (7)	0.0163 (5)	0.0163 (5)	0.0007 (5)	−0.0025 (4)	−0.0011 (4)
C8	0.0597 (9)	0.0205 (6)	0.0241 (6)	−0.0090 (6)	−0.0021 (6)	−0.0033 (5)
C9	0.0379 (7)	0.0204 (6)	0.0178 (5)	0.0002 (5)	−0.0058 (5)	−0.0004 (4)
C10	0.0474 (8)	0.0301 (7)	0.0195 (5)	0.0118 (6)	−0.0011 (5)	−0.0064 (5)
C11	0.0242 (6)	0.0301 (6)	0.0194 (5)	0.0025 (5)	−0.0021 (4)	−0.0005 (4)
C12	0.0289 (7)	0.0346 (7)	0.0434 (8)	−0.0081 (6)	−0.0053 (6)	0.0044 (6)
C13	0.0378 (7)	0.0374 (7)	0.0263 (6)	−0.0042 (6)	−0.0118 (5)	0.0057 (5)
C14	0.0272 (7)	0.0746 (12)	0.0327 (7)	0.0171 (7)	−0.0063 (6)	−0.0116 (7)
C15	0.0276 (6)	0.0184 (5)	0.0215 (5)	−0.0020 (4)	0.0035 (4)	−0.0001 (4)
C16	0.0245 (6)	0.0314 (7)	0.0377 (7)	0.0021 (5)	0.0075 (5)	−0.0009 (5)
C17	0.0192 (6)	0.0355 (7)	0.0334 (6)	−0.0014 (5)	0.0037 (5)	−0.0032 (5)

### Geometric parameters (Å, °)

**Table:**

O1—C1	1.3549 (14)	C9—H9C	0.9800
O1—H1	0.927 (16)	C10—H10A	0.9800
N1—C15	1.2749 (15)	C10—H10B	0.9800
N1—C16	1.4638 (15)	C10—H10C	0.9800
C1—C2	1.4058 (16)	C11—C14	1.5257 (18)
C1—C6	1.4147 (15)	C11—C12	1.5325 (18)
C2—C3	1.3939 (16)	C11—C13	1.5332 (16)
C2—C7	1.5398 (14)	C12—H12A	0.9800
C3—C4	1.4028 (15)	C12—H12B	0.9800
C3—H3	0.9500	C12—H12C	0.9800
C4—C5	1.3863 (15)	C13—H13A	0.9800
C4—C11	1.5328 (16)	C13—H13B	0.9800
C5—C6	1.3914 (16)	C13—H13C	0.9800
C5—H5	0.9500	C14—H14A	0.9800
C6—C15	1.4613 (15)	C14—H14B	0.9800
C7—C10	1.5295 (17)	C14—H14C	0.9800
C7—C8	1.5371 (17)	C15—H15	0.9500
C7—C9	1.5380 (15)	C16—C17	1.5244 (18)
C8—H8A	0.9800	C16—H16A	0.9900
C8—H8B	0.9800	C16—H16B	0.9900
C8—H8C	0.9800	C17—C17^i^	1.525 (3)
C9—H9A	0.9800	C17—H17A	0.9900
C9—H9B	0.9800	C17—H17B	0.9900
			
C1—O1—H1	107.4 (10)	H10A—C10—H10C	109.5
C15—N1—C16	118.61 (11)	H10B—C10—H10C	109.5
O1—C1—C2	120.20 (10)	C14—C11—C12	108.67 (12)
O1—C1—C6	119.71 (10)	C14—C11—C4	112.44 (10)
C2—C1—C6	120.09 (10)	C12—C11—C4	109.40 (10)
C3—C2—C1	117.05 (10)	C14—C11—C13	108.51 (11)
C3—C2—C7	121.48 (10)	C12—C11—C13	108.45 (10)
C1—C2—C7	121.47 (10)	C4—C11—C13	109.28 (10)
C2—C3—C4	124.44 (10)	C11—C12—H12A	109.5
C2—C3—H3	117.8	C11—C12—H12B	109.5
C4—C3—H3	117.8	H12A—C12—H12B	109.5
C5—C4—C3	116.69 (10)	C11—C12—H12C	109.5
C5—C4—C11	120.36 (10)	H12A—C12—H12C	109.5
C3—C4—C11	122.95 (10)	H12B—C12—H12C	109.5
C4—C5—C6	121.73 (10)	C11—C13—H13A	109.5
C4—C5—H5	119.1	C11—C13—H13B	109.5
C6—C5—H5	119.1	H13A—C13—H13B	109.5
C5—C6—C1	120.00 (10)	C11—C13—H13C	109.5
C5—C6—C15	118.70 (10)	H13A—C13—H13C	109.5
C1—C6—C15	121.27 (10)	H13B—C13—H13C	109.5
C10—C7—C8	107.48 (10)	C11—C14—H14A	109.5
C10—C7—C9	107.51 (10)	C11—C14—H14B	109.5
C8—C7—C9	110.11 (10)	H14A—C14—H14B	109.5
C10—C7—C2	111.77 (10)	C11—C14—H14C	109.5
C8—C7—C2	110.79 (9)	H14A—C14—H14C	109.5
C9—C7—C2	109.11 (9)	H14B—C14—H14C	109.5
C7—C8—H8A	109.5	N1—C15—C6	122.40 (10)
C7—C8—H8B	109.5	N1—C15—H15	118.8
H8A—C8—H8B	109.5	C6—C15—H15	118.8
C7—C8—H8C	109.5	N1—C16—C17	110.12 (11)
H8A—C8—H8C	109.5	N1—C16—H16A	109.6
H8B—C8—H8C	109.5	C17—C16—H16A	109.6
C7—C9—H9A	109.5	N1—C16—H16B	109.6
C7—C9—H9B	109.5	C17—C16—H16B	109.6
H9A—C9—H9B	109.5	H16A—C16—H16B	108.1
C7—C9—H9C	109.5	C16—C17—C17^i^	113.31 (13)
H9A—C9—H9C	109.5	C16—C17—H17A	108.9
H9B—C9—H9C	109.5	C17^i^—C17—H17A	108.9
C7—C10—H10A	109.5	C16—C17—H17B	108.9
C7—C10—H10B	109.5	C17^i^—C17—H17B	108.9
H10A—C10—H10B	109.5	H17A—C17—H17B	107.7
C7—C10—H10C	109.5		

Symmetry codes: (i) −*x*+1, −*y*+2, −*z*.

### Hydrogen-bond geometry (Å, °)

**Table:**

*D*—H···*A*	*D*—H	H···*A*	*D*···*A*	*D*—H···*A*
O1—H1···N1	0.927 (16)	1.735 (17)	2.5840 (13)	150.8 (14)
C8—H8B···O1	0.98	2.29	2.9546 (16)	125.
C9—H9A···O1	0.98	2.44	3.0720 (15)	122.
